# Serum and Pleural Soluble Cell Adhesion Molecules in Mesothelioma Patients: A Retrospective Cohort Study

**DOI:** 10.3390/cancers14122825

**Published:** 2022-06-08

**Authors:** Sofia Tsagkouli, Ioannis G. Kyriakoulis, Konstantinos G. Kyriakoulis, Eleni Fyta, Alexandros Syrigos, Petros Bakakos, Adrianni Charpidou, Elias Kotteas

**Affiliations:** 1 Third Department of Medicine, School of Medicine, Sotiria Hospital, National and Kapodistrian University of Athens, 11527 Athens, Greece; stsagouli@yahoo.gr (S.T.); konkyriakoulis@gmail.com (K.G.K.); elenifita1@gmail.com (E.F.); ksyrigos@med.uoa.gr (A.S.); dcharpidou@yahoo.gr (A.C.); ilkotteas@hotmail.com (E.K.); 2 First Department of Chest Medicine, School of Medicine, Sotiria Hospital, National and Kapodistrian University of Athens, 11527 Athens, Greece; petros44@hotmail.com

**Keywords:** mesothelioma, pleural, sE-cadherin, sE-selectin, serum, sICAM-1, soluble cell adhesion molecules, sVCAM-1

## Abstract

**Simple Summary:**

Mesothelioma is an aggressive tumor of mesothelial cells with poor prognosis and limited therapeutic options. Evaluation of the role of well-described molecules would introduce new approaches for prognosis assessment and clinical management in mesothelioma. More importantly, it would pave the way for the development of new, potentially more beneficial therapeutic strategies. In this study, levels of serum and pleural soluble cell adhesion molecules (sCAMs) were measured in patients with malignant pleural mesothelioma. Endpoints that were assessed were: (i) the association of sCAM levels with clinicopathological characteristics of included patients, (ii) the prognostic significance of sCAM levels and (iii) the difference of serum sCAM levels in mesothelioma patients vs. healthy controls. The findings of this study along with future research may contribute to the optimal management of mesothelioma patients.

**Abstract:**

Mesothelioma, a malignant neoplasm of mesothelial cells, has overall poor prognosis. Cell adhesion molecules (CAMs) are proteins that contribute to the immune response. In this study the clinical utility and prognostic significance of serum and pleural fluid soluble CAM (sCAM) levels were assessed in patients with mesothelioma. Mesothelioma patients were retrospectively recruited (2016–2020). Clinical characteristics, serum and pleural sCAM levels (sE-cadherin, sE-selectin, intercellular adhesion molecule 1 (sICAM-1) and vascular cell adhesion molecule 1 (sVCAM-1)) and histopathological characteristics were gathered. A total of 51 healthy controls were also recruited for a secondary cross-sectional analysis. 92 mesothelioma patients were analyzed (mean age 64.5 years, 87% males, performance status 0–2). Patients with increased pleural sE-cadherin had higher risk for disease progression (adjusted HR 1.11 (1.02, 1.20), *p* = 0.013). Serum and pleural sE-selectin were decreased in patients with high-grade mesothelioma. Patients with increased serum or pleural sE-selectin levels had lower risk for death (adjusted HR 0.88 (0.81, 0.96), *p* = 0.003; 0.90 (0.82, 0.99), *p* = 0.039, respectively). Serum sE-cadherin, sE-selectin and sICAM-1 levels were significantly increased in mesothelioma patients compared to healthy controls. Further studies are needed to indicate the clinical utility of serum and pleural sCAMs in mesothelioma patients.

## 1. Introduction

Mesothelioma is a malignant neoplasm of mesothelium—the thin layer of mesodermal-origin mesothelial cells that lines different body cavities [1]. Pleura, peritoneum and pericardium consist of mesothelium and are the most common sites of mesothelioma development [1]. Mesothelioma is a fatal neoplasm with poor prognosis strongly associated with prior asbestos exposure [2]; the median survival from the time of presentation is 12.6 months [3]. The therapeutic approaches include mainly chemotherapy and radiation therapy, whereas recently immunotherapy has been added as a therapeutic option [4]. Surgical intervention is rarely implemented [5]. Unfortunately, none of the above provide a definite cure [2]. Therefore, different therapeutic approaches that may offer better outcomes and survival rates are crucial for the future management of this neoplasm.

Cell adhesion molecules (CAMs) are proteins expressed in the cell surface and play an important role in the binding of cells with other cells or with the extracellular matrix [6]. Among others, the CAM family encompasses calcium-independent binding molecules, such as the members of the immunoglobulin superfamily (IgSF) (e.g., intercellular adhesion molecules (ICAM-1) and vascular cell adhesion molecules (VCAM-1)) and calcium-dependent binding molecules, such as cadherins and selectins. Selectins’ most prominent action is participating in the initial attachment and rolling of leukocytes in endothelium as part of inflammation and leukocyte recruitment [7,8]. Cadherins are primarily involved in cell–cell interactions and maintenance of normal architecture in solid tissues [9]. ICAM-1 and VCAM-1 have important roles in inflammation and immune response [10]. Since the measurement of cellular expression of CAMs is inconvenient in clinical settings, soluble forms of these cell adhesion molecules in the serum have been studied thoroughly.

CAMs are not only greatly involved in the immune response to neoplasms, but also in the pathogenesis of tumor growth and metastasis [10,11,12]. Increasing evidence reveals a linkage between the alterations in adhesion properties of malignant cells and the tumor progression and metastatic potential. Changes in CAM expression provide the neoplastic cell with a migratory phenotype, allowing the cells to perform processes such as loss of epithelial integrity and polarity, intravasation and survival into the bloodstream, as well as colonization in other organs [13]. Soluble forms of these molecules are shown to be relatively increased in a wide variety of malignancies and, in some cases, are associated with the disease severity [14,15,16]. In this context, the aim of our study was to evaluate the levels of different soluble CAMs (sCAMs) in the pleural fluid and serum of patients with mesothelioma and examine possible associations with patients’ demographics, clinical characteristics, disease progression and mortality. Additionally, the difference of sCAM levels between patients and healthy controls was assessed.

## 2. Materials and Methods

### 2.1. Study Design and Setting

A retrospective cohort study was conducted in the reference Oncology Department of the Third Department of Medicine, Sotiria Hospital for Thoracic Diseases, University of Athens, Greece during the period of January 2016–December 2020. The study results were reported in accordance with the Strengthening the Reporting of Observational Studies in Epidemiology (STROBE) statement for cohort, case-control and cross-sectional studies [17]. Ethical approval for this study was obtained from the Sotiria Hospital Scientific Committee. Patient consent was waived due to the retrospective nature of the study.

### 2.2. Participants

Consecutive patients with mesothelioma were recruited. Eligible patients were adults ≥18 years old diagnosed with malignant pleural mesothelioma (histologically or cytologically) who received standard-of-care treatment from the oncology unit. A cohort of 51 healthy control subjects was also recruited for a secondary cross-sectional analysis. Patients and healthy controls that presented signs of infection or leukocytosis were excluded from the study.

### 2.3. Data Collection and Measurement of Variables

Patient clinical data including demographics, treatment and outcome data were obtained from medical records. sCAM levels that were investigated during the study were: serum and pleural soluble (i) E-selectin, (ii) E-cadherin, (iii) ICAM-1 and (iv) VCAM-1. Peripheral venous blood samples were collected into sterile plastic tubes with ethylenediamine tetraacetate in the morning (7.30–9.00 am) after an overnight 12 h fast. Pleural samples were similarly collected in the morning depending on thoracic surgeons’ availability. Samples were centrifuged at 3000 rpm for 20 min (within 2 h from collection), and serum was separated, aliquoted and stored at −80 °C until assay. sCAMs concentrations were determined using a solid phase, enzyme-linked immunosorbent assay (ELISA). sE-cadherin was measured using a special immunoassay kit (Human Elisa sE-cadherin BioVendor, Brno, Czech Republic), (sensitivity of 0.5 ng/mL). In the same manner, sE-selectin, sICAM-1 and sVCAM-1 were measured using HUMAN E-SELECTIN ELISA Life Technologies, CA, USA, sensitivity of 0.13 ng/mL; Human Elisa sICAM-1 BioVendor, Brno, Czech Republic, sensitivity of 0.2 ng/mL; and Human Elisa VCAM-1 BioVendor, Brno, Czech Republic, sensitivity of 0.2 ng/mL, respectively.

### 2.4. Outcomes

The primary outcomes of the study were (i) the association of serum and pleural sCAM levels with patients’ baseline demographics and clinical characteristics, (ii) the association between serum and pleural sCAM levels and disease progression, (iii) the association between serum and pleural sCAM levels and mortality, and (iv) the assessment of differences of serum sCAM levels among patients and healthy controls.

### 2.5. Statistical Analysis

The normality of continuous quantitative variables was assessed using the Kolmogorov–Smirnov test. Mean (standard deviation (SD)) and median (interquartile range (IQR)) values were used to describe quantitative variables. Absolute values and respective frequencies (%) were used to describe continuous variables. Comparison of quantitative variables between subgroups was performed with an independent sample *t*-test, whereas comparison between qualitative variables was performed with a chi-squared or Fisher’s exact test, as appropriate. The linear correlation between quantitative variables was assessed using Pearson’s or Spearman’s rank correlation coefficient (r), as appropriate. The correlation was interpreted as weak (r = 0.1–0.3), moderate (r = 0.31–0.50) and strong (r > 0.50). Univariate and multivariate Cox regression analyses were performed in order to calculate hazard ratios (HRs) (95% confidence intervals (CI)) and identify determinants of the cumulative incidence of the endpoints of interest (disease progression and mortality). Unadjusted (uHR) and adjusted (aHR) HRs were calculated and the respective Kaplan–Meier curves were plotted. HRs for CAM levels were expressed for 10 units (ng/mL) of increase. Linear regression models were used to investigate the difference between CAM levels between mesothelioma patients and healthy controls after adjustment for gender, age and smoking habit. The statistical package IBM SPSS Statistics for Windows, Version 22.0 (IBM Corp, Armonk, NY, USA) was used. Statistical significance was set at a 0.05 level.

## 3. Results

### 3.1. Subjects’ Baseline Characteristics

The study cohort consisted of 92 mesothelioma patients. Mean age (SD) was 64.5 (9.2) years, 80 patients (87%) were males and 40 (44%) had previous asbestos exposure. The majority of patients had an epithelial histological type of mesothelioma (77.1%) and almost half of all mesotheliomas were high grade. All patients were receiving chemotherapeutic agents and 15 of them also had radiation therapy. The baseline demographics and clinical characteristics of included patients are reported in Table 1. For the evaluation of differences of sCAM levels in mesothelioma and non-mesothelioma subjects, a second cohort of 51 healthy control subjects (age 60.7 (8.7) years, 51 (85%) males and 52 (87%) smokers) was recruited. Serum sCAM levels of patients and controls and pleural sCAM levels of patients are reported in Table 2. Due to the retrospective nature of this study, a post-hoc analysis (assuming a level of significance of 0.05, and sample size n = 92 patients and n = 51 controls) was performed that confirmed adequate power in demonstrating differences in sCAM levels between patients and controls (power > 0.80). The association between different patients’ baseline and follow-up characteristics with disease progression and mortality are reported in Table S1/Figure S1 and in Table S2/Figure S2, respectively.

### 3.2. sE-Cadherin

**Figure 1:**
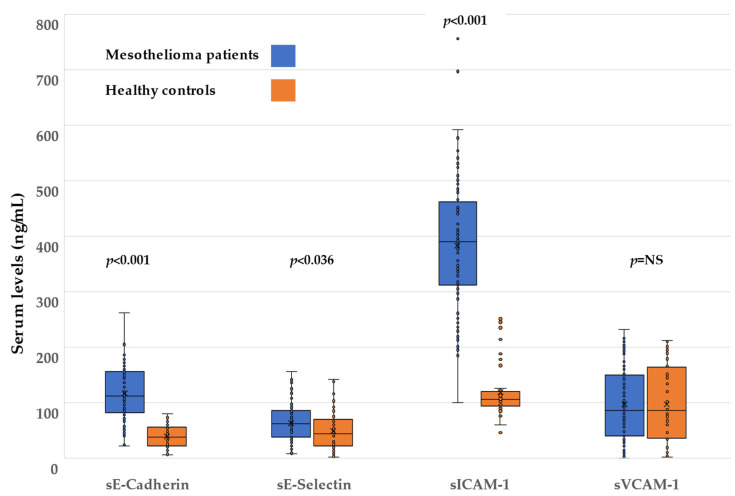
Box plots demonstrating sCAM levels in mesothelioma patients vs. healthy controls; “x” corresponds to mean values (see also Table 2). NS, non-significant.

Pleural sE-cadherin was found to be significantly higher in men than women (mean (SD) 116.1 (49.6) vs. 82.3 (34.1) ng/mL, *p* = 0.026). sΕ-cadherin serum levels were positively associated with age; older patients had significantly higher levels (correlation coefficient r = 0.22, *p* = 0.039). In the unadjusted analysis, patients with increased pleural sE-cadherin levels (uHR 1.08 (1.01, 1.17), *p* = 0.043) were found to have statistically significant higher risk for disease progression. In the multivariate analysis (adjustment for gender, age, smoking, PS, stage of tumor and tumor grade), patients with increased pleural sE-cadherin levels presented statistically significant higher risk for disease progression (aHR 1.11 (1.02, 1.20), *p* = 0.013). No association was demonstrated between serum or pleural sE-cadherin levels and mortality. After adjustment for gender, age and smoking habit, sE-cadherin serum levels were significantly higher (difference = 78.8 ng/mL) in patients compared with healthy controls (*p* < 0.001) (Figure 1).

### 3.3. sE-Selectin

sE-selectin levels, both in serum and pleural fluid, were significantly different depending on the grade of the tumor; patients with a high-grade tumor had lower sE-selectin levels compared with patients with low-grade mesothelioma (serum 52.7 (33.33) vs. 75.8 (29) ng/mL, *p* = 0.01 and pleural 56.8 (31.3) vs. 71.2 (29.7) ng/mL, *p* = 0.033). In the multivariate analysis (adjustment for gender, age, smoking, asbestos exposure and tumor stage) both serum and pleural increased sE-selectin levels were found to be associated with lower risk for death: aHR 0.88 (0.81, 0.96), *p* = 0.003 and 0.90 (0.82, 0.99), *p* = 0.039, respectively. After adjustment for gender, age and smoking habit, sE-selectin serum levels were significantly higher (difference = 13.9 ng/mL) in patients compared with healthy controls (*p* < 0.036) (Figure 1).

### 3.4. sICAM-1

sICAM-1 levels did not show correlation with disease progression or mortality in both univariate and multivariate analyses. After adjustment for gender, age and smoking habit, sICAM-1 serum levels were significantly higher (difference = 265.6 ng/mL) in patients compared with healthy controls (*p* < 0.001) (Figure 1).

### 3.5. sVCAM-1

sVCAM-1 levels in pleural fluid were significantly lower in patients who had dyspnea as the initial presenting symptom compared to those who did not (77.1 (59.8) vs. 105.1 (72.8) ng/mL, *p* = 0.05). Serum sVCAM-1 levels did not show any significant difference between patients and controls (*p* = NS) (Figure 1). sVCAM-1 levels did not show correlation with disease progression or mortality in both univariate and multivariate analyses.

### 3.6. Correlation between Serum and Pleural sCAM Levels in Patients

**Figure 2:**
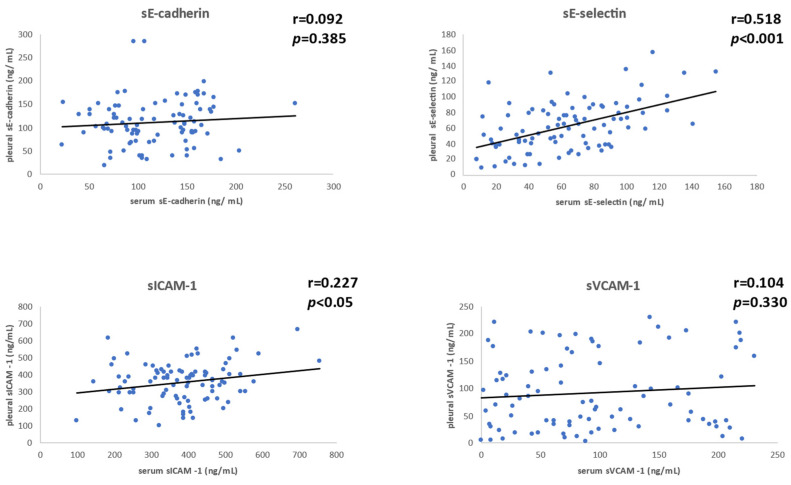
Scatter plots presenting the relationships between serum and pleural sCAM levels in mesothelioma patients.

Pleural sE-cadherin and sVCAM-1 were not correlated with serum sE-cadherin and sVCAM-1 levels (r = 0.092, *p* = 0.385 and r = 0.104, *p* = 0.330, respectively). A weak correlation was found between pleural and serum sICAM-1 (r = 0.227, *p* < 0.05). Finally, serum and pleural sE-selectin levels were found to be strongly correlated (r = 0.518, *p* < 0.001). Scatter plots showing correlation between serum and pleural sCAM levels in patients are presented in Figure 2.

## 4. Discussion

The main findings of our study were: (i) elevated pleural sE-cadherin was associated with higher risk of disease progression and significantly higher levels of serum sE-cadherin were found in mesothelioma patients, (ii) elevated serum and pleural sE-selectin was associated with lower mortality risk and significantly higher levels of serum sE-selectin were found in mesothelioma patients, (iii) significantly higher levels of serum sICAM-1 were found in mesothelioma patients, and (iv) no correlation between disease progression or mortality and sVCAM-1 levels and no significant difference in sVCAM-1 levels between patients and controls were found.

To the best of our knowledge, this is the first study assessing and comparing serum and pleural sE-cadherin, sE-selectin, sICAM-1 and sVCAM-1 in healthy subjects vs. malignant mesothelioma patients.

Serum sE-cadherin was found to be significantly elevated in mesothelioma patients compared to healthy controls in our cohort. No relevant data were identified in the literature concerning the role of serum sE-cadherin in malignant mesothelioma. However, these results are in line with previous findings in other types of malignancy. In lung cancer (including non-small-cell lung cancer (NSCLC) (squamous cell carcinoma (SCC) and adenocarcinoma) and small-cell lung cancer (SCLC)), increased sE-cadherin levels have been found in patients relative to healthy controls. Cioffi et al. performed sE-cadherin level measurements in 79 lung cancer patients and 52 non-lung cancer subjects (9 with breast cancer, 10 with benign pulmonary disease, 13 with non-pulmonary disease and 20 healthy controls). Using a cutoff of 2.56 μg/mL and after achieving a specificity level of 90%, Cioffi et al. demonstrated a sensitivity of 66.6%, 47.6% and 43.7% for diagnosing SCC of the lung, SCLC and adenocarcinoma, respectively [18]. Additionally, data from colorectal carcinoma [19], gastric cancer, breast cancer and bladder cancer patients reveal prominent sE-cadherin elevation in cancer patients relative to healthy controls, along with worse outcomes [20,21]. Interestingly, in a study with 166 gastric cancer patients and 71 healthy controls, Juhasz et al. found two different patterns of fluctuation of sE-cadherin; significantly higher sE-cadherin in intestinal-type gastric cancer, but significantly lower sE-cadherin in diffuse-type gastric cancer [22]. In our study, patients with increased sE-cadherin levels in pleural fluid presented higher risk for disease progression both in univariate and multivariate analyses. In another study, Charalabopoulos et al. reported that increased serum sE-cadherin was associated with distant metastasis and worse prognosis in patients with NSCLC [23]. On a cellular level, E-cadherin downregulation has been proved to be associated with epithelial-mesenchymal transformation (ΕΜΤ), a process of functional, cytological transition of cancer cells with epithelial properties to a mesenchymal cell phenotype with more aggressive survival and spread properties [24]. EMT can also be seen in mesothelioma and leads to histopathological features (sarcomatoid or biphasic mesothelioma) that are associated with worse prognosis [25]. In a tissue microarray study on 14,637 tumor samples from 112 different tumor types—including mesothelioma—the inversion of the E-cadherin expression pattern (downregulation of E-cadherin in cancers derived from E-cadherin positive tissues and upregulation of E-cadherin in cancers derived from E-cadherin negative tissues) was linked to aggressive histopathological tumor types and poor outcome [26].

In our study, mesothelioma patients had increased serum sE-selectin compared to controls. Previous data concerning other malignancies are in agreement with these observations. More specifically, with regards to lung cancer (SCLC and NSCLC), increased serum sE-selectin levels have been observed in patients relative to healthy controls [27,28]. This difference in sE-selectin levels between patients and healthy controls has also been identified in patients with breast cancer [29,30,31,32], colorectal cancer [15,33] and gastric cancer [34]. Mesothelioma patients with increased serum and pleural sE-selectin had better rates of survival in our cohort. However, in other types of malignancy, increased sE-selectin concentrations have been found to correlate with poor prognosis [33,34]. E-selectins are greatly involved in acute and chronic immune response and play an important role in mediating cell–cell adhesions and the migration of leukocytes and cancer cells to the surrounding tissues [7,8]. E-selectin expression in endothelial cells has been found to be upregulated during metastatic processes [35,36]. Interestingly, in a study involving patients with colorectal cancer, the authors concluded that increased levels of sE-selectin may indicate liver metastasis [34]. sE-selectin has also been found to accelerate the process of migration and infiltration of leucocytes and cancer cells in breast cancer [31]. Furthermore, sE-selectin has been characterized as a mediator of angiogenesis, an important aspect of tumorigenesis [37].

Our findings demonstrate a great increase in sICAM-1 in mesothelioma patients compared to controls. Elevated sICAM-1 probably reflects the increased immune response activation against malignant mesothelioma, considering its distinct important role in the immune response process [38]. Furthermore, the rise of sICAM-1 may be attributable to the complex pathogenesis and survival of the tumor, since ICAM-1 is considered to be of great significance in terms of tumorigenesis [38]. More importantly, ICAM-1 contributes to the transendothelial tumor cell migration process and, thus, it has an active role in the metastatic potential of the tumor [12]. Increased sICAM-1 concentration has been found in pleural effusions caused by mesotheliomas, NSCLC and gynecologic malignancies [39]. There are many studies linking increased sICAM-1 with the diagnosis and prognosis of a variety of malignancies. In a recent large meta-analysis including 23 observational studies, serum sICAM-1 in patients with SCLC and NSCLC was significantly higher in patients than controls, and higher sICAM-1 levels were significantly correlated with worse prognosis [40]. Overall, authors stated that sICAM-1 may serve as a potential biomarker for diagnosing lung cancer and predicting its stage and prognosis. Bulska-Bedkowska et al. studied 39 women with advanced breast cancer and reported the usefulness of serum sICAM-1 and sVCAM-1 in predicting overall survival and progression-free survival time, respectively [14]. Pospelova et al. demonstrated significant association between elevated sICAM-1 and sPECAM-1 (another important CAM not assessed in the present study) serum levels and central nervous system complications in women following breast cancer treatment [41]. In a study including 57 gastric cancer patients, significant elevation of sICAM-1 was observed compared to healthy controls. Interestingly, this relative elevation disappeared after tumor resection was performed. Moreover, higher levels of sICAM-1 were found to correlate with worse cancer-related outcomes [16]. Finally, relatively increased sICAM-1 levels were also found in studies with colorectal carcinoma patients [15,42].

sVCAM-1 was the only sCAM measured in our study, the levels of which did not differ significantly between patients and controls. A significant difference of sVCAM-1 in patients relative to healthy controls has been reported previously in other types of malignancy, such as lung and colorectal cancer [15,43]. This discrepancy could be attributed to different baseline characteristics between patients and controls in our study (smokers 54.3% vs. 86.7%, respectively, *p* < 0.001) that have been shown to potentially influence sVCAM-1 levels [44]. VCAM-1 is a molecule, expressed mostly on the surface of endothelial cells, with a well-characterized role in inflammation (leukocyte adhesion and diapedesis) and immune response [10]. Recent data indicate a relationship between VCAM-1 expression and cancer metastasis and angiogenesis [45].

The shedding of CAMs from the cell membrane and release of their soluble forms in the serum is performed by a wide variety of enzymes (sheddases). Therefore, levels of soluble forms of CAMs are also affected by the activity of these enzymes. This ectodomain cleavage is thought to interfere with the interaction between leukocytes and endothelial cells during the process of leukocyte recruitment [46].

The findings of the present study should be interpreted taking into consideration important limitations. First, the retrospective nature of the study constitutes a certain limitation. In addition, 15 patients received radiation therapy. It has been previously described that radiation therapy interferes with CAM expression and function and, consequently, interferes with tumor metastasis, angiogenesis, inflammation and immune response [47]. This interfering may constitute a component of radiation-induced tumor control and it is unknown to what extent it has affected our findings. Moreover, asbestos exposure was evaluated by patients’ medical records and clinical history. It is unknown if some patients were unaware of their previous exposure. In this way, an underestimation of the number of patients with previous asbestos exposure cannot totally be eliminated.

## 5. Conclusions

In conclusion, the association of clinical features, disease progression and mortality of mesothelioma patients with the concentrations of sCAMs is not yet clearly defined. Although sCAM levels can offer valuable hints, their clinical contribution and advantages in the management of patients with mesothelioma is a matter of discussion. Basic science studies, as well as prospective randomized clinical trials with a purposeful approach, are needed to shed light on the potential clinical usefulness of these biomarkers in mesothelioma.

## Figures and Tables

**Table 1:** Baseline characteristics of included mesothelioma patients.

Characteristics		Patients
N		92
Gender	Male, *no.* (%)	80 (87.0)
	Female, *no.* (%)	12 (13.0)
Age, mean (SD)		64.5 (9.2)
Smokers, *no.* (%)		50 (54.3)
Pack-years, mean (SD)		54.1 (23.6)
Asbestos exposure, *no.* (%)		40 (44)
Weight loss > 10%, *no.* (%)		19 (20.7)
Initial presenting symptom	Cough, *no.* (%)	12 (13)
	Dyspnea, *no.* (%)	26 (28.3)
	Thoracic pain, *no.* (%)	24 (26.1)
	Other or mixed initial symptoms, *no.* (%)	30 (32.6)
Performance Status	0, *no.* (%)	43 (46.7)
	1, *no.* (%)	40 (43.5)
	2, *no.* (%)	9 (9.8)
Stage	Ι, *no.* (%)	42 (45.7)
	ΙΙ, *no.* (%)	6 (6.5)
	ΙΙΙ, *no.* (%)	11 (12)
	IV, *no.* (%)	33 (35.9)
Histologic type	Epithelial/epithelioid, *no.* (%)	71 (77.1)
	Desmoplastic, *no.* (%)	4 (4.3)
	Sarcomatoid, *no.* (%)	11 (11.9)
	Other, *no.* (%)	6 (6.5)
Grade	High, *no.* (%)	42 (49.4)
	Low, *no.* (%)	43 (50.6)
Radiation therapy	Yes, *no.* (%)	15 (19.2)
	No, *no.* (%)	63 (80.8)

**Table 2:** Serum sCAM levels of patients and controls and pleural sCAM levels of patients.

	sCAMs	Patients [mean (SD), ng/mL]	Controls [mean (SD), ng/mL]	*p*
Serum	sE-cadherin	117.5 (44.4)	38.7 (20)	**<0.001**
	sE-selectin	63.2 (33.1)	49.3 (35.7)	**0.015**
	sVCAM-1	95.6 (68.4)	96.6 (69.2)	0.927
	sICAM-1	383.7 (117)	118.1 (44.7)	**<0.001**
Pleural	sE-cadherin	111.6 (49.1)	-	-
	sE-selectin	62.1 (31.4)	-	-
	sVCAM-1	92.2 (68.2)	-	-
	sICAM-1	356.6 (116.3)	-	-

## Data Availability

The data that support the findings of this study are available from the corresponding author (I.G.K.) upon reasonable request.

## Supplementary Materials

The following supporting information can be downloaded at: https://www.mdpi.com/article/10.3390/cancers14122825/s1, Figure S1: Kaplan–Meier plot demonstrating disease progression per performance status group; Figure S2: Kaplan–Meier plot demonstrating mortality per performance status group; Table S1: Disease progression relative to demographics, clinical characteristics and serum and pleural soluble cell adhesion molecule levels in 92 mesothelioma patients. (Results derived from univariate analysis); Table S2: Mortality relative to demographics, clinical characteristics and serum and pleural cell adhesion molecule levels in 92 mesothelioma patients. (Results derived from univariate analysis).
