# Supplementary material for: Serum and Pleural Soluble Cell Adhesion Molecules in Mesothelioma Patients: A Retrospective Cohort Study
Source: Cancers (Basel). 2022 Jun 8;14(12):2825. doi: 10.3390/cancers14122825 (PMC9221497; doi:10.3390/cancers14122825)
Supplement: Supplementary file 1 [file cancers-14-02825-s001.zip › cancers-1694002-supplementary.pdf]

Serum and pleural soluble cell adhesion molecules in mesothelioma patients: a retrospective cohort study

SUPPLEMENTARY FILE

Table S1. Disease progression relative to demographics, clinical characteristics and serum and pleural soluble cell adhesion molecules levels in 92 mesothelioma patients. (Results derived from univariate analysis).

**Variables**			
**Baseline characteristics**		*HR (95% CI)*	*P*
Gender	Male (ref)		
	Female	0.90 (0.31-2.62)	0.843
Age		1.01 (0.96-1.05)	0.724
Smoker	No (ref)		
	Yes	0.82 (0.37-1.8)	0.623
Asbestos exposure	No (ref)		
	Yes	0.66 (0.29-1.5)	0.322
Weight loss > 10%	No (ref)		
	Yes	1.74 (0.74-4.06)	0.204
Performance Status		**2.35 (1.33-4.14)**	**0.003**
Stage		1.24 (0.92-1.66)	0.651
**Initial presenting symptom**			
Cough	No (ref)		
	Yes	0.8 (0.3-2.13)	0.651
Dyspnea	No (ref)		
	Yes	2.31 (1.04-5.16)	0.041
Thoracic pain	No (ref)		
	Yes	0.64 (0.29-1.43)	0.281
Grade	High (ref)		
	Low	0.63 (0.26-1.49)	0.291
Radiation Therapy	No (ref)		
	Yes	1.62 (0.58-4.54)	0.361
**Cancer location and spread on time of diagnosis**			
Pleura	No (ref)		
	Right	0.36 (0.05-2.78)	0.328
	Left	0.37 (0.05-3.04)	0.357
	Bilateral	0.3 (0.03-3.42)	0.336
Pericardium	No (ref)		
	Yes	0.96 (0.29-3.22)	0.951
Peritoneum	No (ref)		
	Yes	1.55 (0.36-6.6)	0.555
Bones	No (ref)		
	Yes	1.36 (0.53-3.45)	0.519
Lymph nodes	No (ref)		
	Yes	1.31 (0.52-3.3)	0.563
Lung	No (ref)		
	Yes	1.18 (0.53-2.6)	0.686
Liver	No (ref)		
	Yes	2.44 (0.72-8.3)	0.154
Another organ	No (ref)		

	Yes	1.03 (0.24-4.37)	0.969
**Cancer location and spread on follow up**			
Pleura	No (ref)		
	Yes	21.16 (0-299.79)	0.614
Pericardium	No (ref)		
	Yes	0.80 (0.18-3.5)	0.767
Peritoneum	No (ref)		
	Yes	0.04 (0.00-21.17)	0.315
Bones	No (ref)		
	Yes	0.82 (0.29-2.28)	0.705
Lymph nodes	No (ref)		
	Yes	**3.99 (1.49-10.66)**	**0.006**
Lung	No (ref)		
	Yes	**2.91 (1.02-8.3)**	**0.045**
Liver	No (ref)		
	Yes	1.54 (0.5-4.72)	0.453
Brain	No (ref)		
	Yes	5.69 (0.73-44.46)	0.097
Other organ	No (ref)		
	Yes	0.04 (0.0-84.54)	0.416
**Soluble cell adhesion molecules serum and pleural levels (ng/ml)**		*HR (95% CI)**	*P*
sE-CADHERIN serum		0.98 (0,9-1.07)	0.644
sE-CADHERIN pleural		**1.08 (1,01-1.17)**	**0.043**
sE-SELECTIN serum		0.92 (0,81-1.04)	0.193
sE-SELECTIN pleural		0.96 (0,84-1.09)	0.514
sICAM serum		0.99 (0,96-1.03)	0.6
sICAM pleural		1.02 (0,98-1.05)	0.343
sVCAM serum		1.00 (0,95-1.06)	0.884
sVCAM pleural		0.96 (0.89-1.02)	0.197

HR (95% CI), hazard ratio (95% confidence intervals); sICAM, soluble intercellular adhesion molecules; ref, reference; sVCAM, soluble vascular cell adhesion molecules

*per 10 units of increase

Table S2. Mortality relative to demographics, clinical characteristics and serum and pleural cell adhesion molecules levels in 92 mesothelioma patients. (Results derived from univariate analysis).

**Variables**			
**Baseline characteristics**		*HR (95% CI)*	*P*
Gender	Male (ref)		
	Female	0.65 (0.33-1.27)	0.204
Age		1.02 (0.99-1.04)	0.197
Smoker	No (ref)		
	Yes	1.31 (0.79-2.15)	0.293
Asbestos exposure	No (ref)		
	Yes	1.05 (0.63-1.73)	0.861
Weight loss > 10%	No (ref)		
	Yes	**2.47 (1.37-4.47)**	**0.003**
Performance Status		**2.47 (1.64-3.72)**	**<0.001**
Stage		**1.36 (1.13-1.62)**	**0.001**
**Initial presenting symptom**			
Cough	No (ref)		
	Yes	0.94 (0.53-1.67)	0.844
Dyspnea	No (ref)		
	Yes	1.06 (0.65-1.75)	0.810
Thoracic pain	No (ref)		
	Yes	1.22 (0.75-1.97)	0.425
Grade	High (ref)		
	Low	**0.56 (0.33-0.94)**	**0.028**
Radiation Therapy	No (ref)		
	Yes	0.67 (0.34-1.32)	0.245
**Cancer location and spread on time of diagnosis**			
Pleura	No (ref)		
	Right	0.23 (0.03-1.79)	0.162
	Left	0.23(0.03-1.77)	0.157
	Bilateral	0.18(0.02-1.58)	0.121
Pericardium	No (ref)		
	Yes	1.5 (0.68-3.32)	0.317
Peritoneum	No (ref)		
	Yes	**4.37 (1.82-10.48)**	**0.001**
Bones	No (ref)		
	Yes	**3.86 (2.04-7.32)**	**<0.001**
Lymph nodes	No (ref)		
	Yes	**2.04 (1.11-3.75)**	**0.022**
Lung	No (ref)		
	Yes	1.1 (0.67-1.79)	0.714
Liver	No (ref)		
	Yes	**8.28 (3.14-21.86)**	**<0.001**
Another organ	No (ref)		
	Yes	**2.67 (1.18-6.02)**	**0.018**
**Cancer location and spread on follow up**			
Pleura	No (ref)		
	Yes	1.04 (0.14-7.61)	0.971
Pericardium	No (ref)		
	Yes	1.07 (0.5-2.29)	0.860
Peritoneum	No (ref)		

	Yes	0.96 (0.41-2.28)	0.933
Bones	No (ref)		
	Yes	1.49 (0.84-2.64)	0.170
Lymph nodes	No (ref)		
	Yes	1.37 (0.77-2.44)	0.291
Lung	No (ref)		
	Yes	0.7 (0.39-1.25)	0.231
Liver	No (ref)		
	Yes	1.22 (0.6-2.48)	0.587
Brain	No (ref)		
	Yes	0.66 (0.09-4.85)	0.684
Other organ	No (ref)		
	Yes	0.77 (0.27-2.17)	0.622
**Soluble cell adhesion molecules serum and pleural levels (ng/ml)**		*HR (95% CI) **	*P*
sE-CADHERIN serum		0.96 (0.91-1.02)	0.168
sE-CADHERIN pleural		1.03 (0.98-1.09)	0.235
sE-SELECTIN serum		0.95 (0.88-1.03)	0.231
sE-SELECTIN pleural		0.94 (0.87-1.02)	0.139
sICAM serum		0.99 (0.96-1.01)	0.235
sICAM pleural		1.01 (0.99-1.03)	0.501
sVCAM serum		1.00 (0.96-1.03)	0.895
sVCAM pleural		0.99 (0.96-1.03)	0.707

HR (95% CI), hazard ratio (95% confidence intervals); sICAM, soluble intercellular adhesion molecules; ref, reference; sVCAM, soluble vascular cell adhesion molecules

*per 10 units of increase

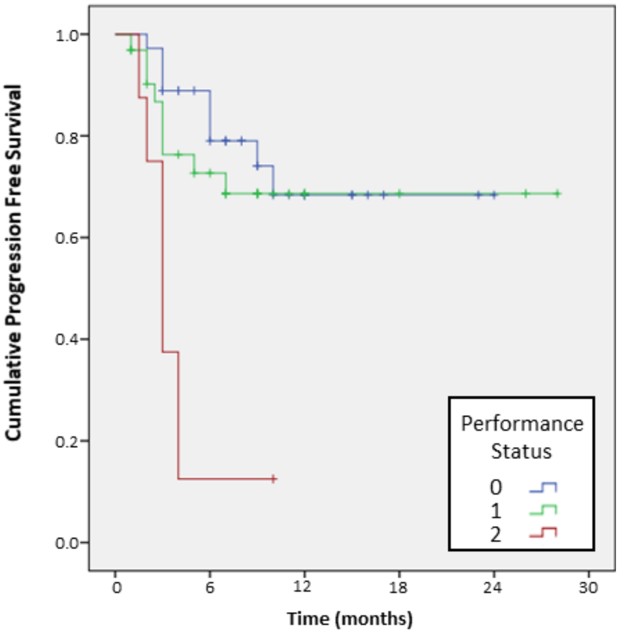

Figure S1. Kaplan-Meier plot demonstrating disease progression per performance status group.

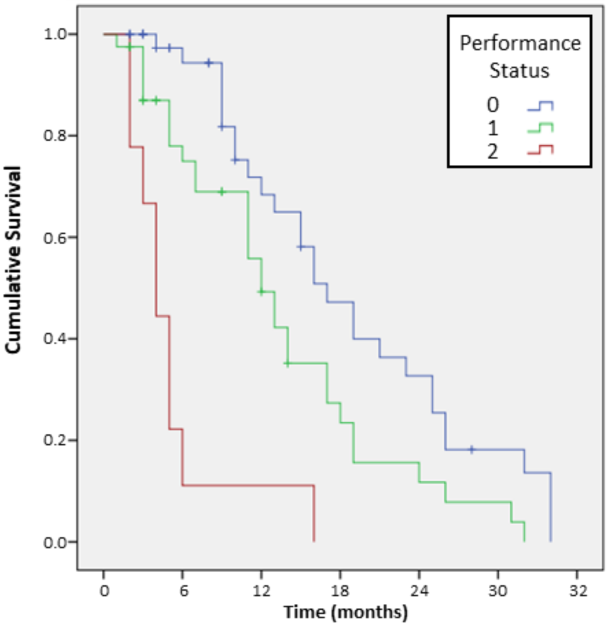

Figure S2. Kaplan-Meier plot demonstrating mortality per performance status group. .
